# High Production of Chitinolytic Activity in Halophilic Conditions by a New Marine Strain of *Clonostachys rosea*

**DOI:** 10.3390/molecules24101880

**Published:** 2019-05-16

**Authors:** Marcella Pasqualetti, Paolo Barghini, Valeria Giovannini, Massimiliano Fenice

**Affiliations:** 1 Dipartimento di Scienze Ecologiche e Biologiche, University of Tuscia, 01100 Viterbo, Italy; mpasqual@unitus.it (M.P.); barghini@unitus.it (P.B.); v.giovannini@unitus.it (V.G.); 2 Laboratorio di Ecologia dei Funghi Marini, CoNISMa, University of Tuscia, 01100 Viterbo, Italy; 3 Laboratorio di Microbiologia Marina Applicata, CoNISMa, University of Tuscia, 01100 Viterbo, Italy

**Keywords:** marine fungi, halophiles, chitinolytic enzymes, high producer, *Clonostachys rosea*, screening, Response Surface Methodology

## Abstract

Twenty-eight fungal strains have been isolated from different natural marine substrates and plate screened for their production of chitinolytic activity. The two apparent best producers, *Trichoderma* *lixii* IG127 and *Clonostachys* *rosea* IG119, were screened in shaken cultures in media containing 1% colloidal chitin, 1% yeast nitrogen base and 38‰ NaCl, for their ability to produce chitinolytic enzymes under halophilic conditions. In addition, they were tested for optimal growth conditions with respect to pH, salinity and temperature. The *Trichoderma* strain appeared to be a slight halotolerant fungus, while *C.* *rosea* IG119 clearly showed to be a halophilic marine fungus, its optimal growth conditions being very coherent for life in the marine environment (i.e., pH 8.0, salinity 38‰). Due to its high and relatively fast activity (258 U/L after 192 h of growth) accompanied by its halophilic behaviour (growth from 0 to 160‰ of salinity), *C.* *rosea* was selected for further studies. In view of possible industrial applications, its medium for chitinolytic enzyme production was optimized by Response Surface Methodology using 1% colloidal chitin and different concentrations of corn step liquor and yeast nitrogen base (0–0.5%). Time course of growth under optimized condition showed that maximum activity (394 U/L) was recorded after 120 h on medium containing Corn Steep Liquor 0.47% and Yeast Nitrogen Base 0.37%. Maximum of productivity (3.3 U/Lh) was recorded at the same incubation time. This was the first study that demonstrated high chitinolytic activity in a marine strain of *C.* *rosea.*

## 1. Introduction

The various and multiform marine environments are sources of chemical and biological diversity of paramount importance and an inexhaustible resource of unexploited and/or unknown microorganisms. Hence, oceans and seas represent a huge reserve of new substances with potential applications in feed and food, fine chemicals, pharmaceutical and enzyme industries [1,2,3]. The industrial and commercial value of enzymes increased in the past two decades in traditional fields such as food and detergent industries, but also in different areas, where it is still growing, particularly in textile and leather industries, environment depollution, medical applications, biotechnology, bioenergy, biosensors and so on. A recent assessment (2017) has valued the global enzymes market at more than $7000 million, and the business is estimated to exceed $10,000 million in 2024 [4]. 

The search of new or improved enzymes is a very worthwhile business for which the marine environment should not be overlooked since there is a large interest in recruiting microbial enzymes for the development of environmentally-friendly industrial applications [1,5]. Interesting new biocatalysts with peculiar properties, such as high salt tolerance, hyperthermostability, piezophilicity and cold adaptivity, can be obtained by marine enzyme biotechnology [6]. Actually, the knowledge of enzymes having optimal activities at uncommon values of salt concentration, pH and temperature is useful; in addition, a marine-derived enzyme may show novel and useful chemical and stereochemical properties [2].

Nevertheless, the exploitation of marine environments for new enzyme producers is still scarce if compared with other environments [1,6]. Only a rather limited number of works have been carried out to screen in detail the marine microorganisms’ capacity to produce extracellular enzymes and end even less investigated marine fungi [1,7,8].

On the whole, the most commonly studied enzymes are those involved in the hydrolysis of biopolymers. Among them, chitinolytic enzymes, even though rather widely studied, still need investigation for their wide pattern of industrial and environmental applications where new and suitable sources of these bio-catalysts are requested [9].

Chitin is one of the most abundant natural polysaccharides; it is an important source of carbon and nitrogen for marine organisms and its turnover in the aquatic biosphere is quite high. It has been estimated that more than 10^11^ metric tons of this polysaccharide are produced annually and the oceans represent a huge resource of chitin that should be transformed in other biological materials to avoid carbon and nitrogen depletion in these environments. Actually, marine sediments contain only trace of chitin, the degradation of which is a main step in the cycling of nutrients in the oceans that is traditionally attributed mainly to bacteria [10,11,12]. In addition, fungi can contribute substantially to chitin degradation and recycle, particularly in some marine environments such as sediments and estuaries [10,13].

Chitin hydrolysis is carried out by a series of chitin-degrading enzymes, the classification of which is quite ambiguous and confusing [9,14]. Anyway, current classification, given by the Enzyme Commission (EC), only considers two classes of enzymes: Chitinase, (EC 3.2.1.14) performing “random hydrolysis of *N*-acetyl-β-d-glucosaminide (1-4)-β-linkages in chitin and chitodextrins”; and β-*N*-acetylhexosaminidase (EC 3.2.1.52) that “releases N-Acetyl-D-hexosamine residues, at the non-reducing terminal, from chitin and chitodextrins”. 

Nonetheless, numerous authors still use old classifications and use different “names” for the various chitinolytic activities. The enzymes performing a random hydrolysis inside the polysaccharide, producing shorter fragments, are often defined as “endo-chitinases”, while those having an external activity are defined as “exo-chitinases”. The enzymes acting only on chitobiose are called “chitobiases”, while those releasing chitobiose from chitin or chitodextrins are named “chitobiosidases” [9,14,15,16,17].

While some innovative applications have been tested at the laboratory level [18,19], these enzymes traditionally are used in the hydrolysis of chitin and chitin-containing materials, production of chitin derivatives, protoplast formation and biocontrol of pathogenic organisms [9]. However, some of the mentioned environmental applications (i.e., on-field control of pathogens or degradation of chitin-rich wastes), can be sturdily limited by adverse conditions, in particular low temperature and salinity, which could affect the microbial activity. It has been demonstrated that psychrotolerant microorganisms, producing high levels of cold-active chitinolytic enzymes, can be important to replace mesophilic strains in case of applications at low temperature: Here the search for new chitinolytic organisms is of great applied interest [9,20,21,22]. By contrast, the search for microorganisms able to produce high levels of chitinolytic enzymes in halophilic conditions is rather scarce and mainly regards bacteria; marine fungi have been somehow overlooked and chitinase production has only been studied in a few species [23,24]. The availability of marine chitinolytic microorganisms and/or enzymes, able to work in halophilic conditions and in broad ranges of other parameters such as pH, would be useful in various fields. For example, they could find applications in the production of high-value chitin-derivatives (i.e., chito-oligosaccharides) or degradation of salty chitin-rich materials (i.e., residues from the crab or shrimp industries). They could be also used in food technology to control spoiling organisms where high concentration of NaCl is present [25,26,27].

In this study, we report on a screening carried out on 28 fungal strains (obtained from various substrates collected in the East Sector of the Tyrrhenian Sea) in order to test their potential to produce high level of extracellular chitinolytic enzymes activity. The activity of most promising strains was verified in shaken cultures and they were investigated for optimal growth conditions with respect to pH, temperature and salinity. Among them, the ascomycete *Clonostachys rosea* IG119, was investigated in details for its high production in view of possible industrials applications; optimization of its culture media to maximize the enzyme production was carried out by Response Surface Methodology (RSM).

## 2. Results and Discussion

### 2.1. Plate Screening for Chitinolytic Enzyme Producers

**Figure 1:**
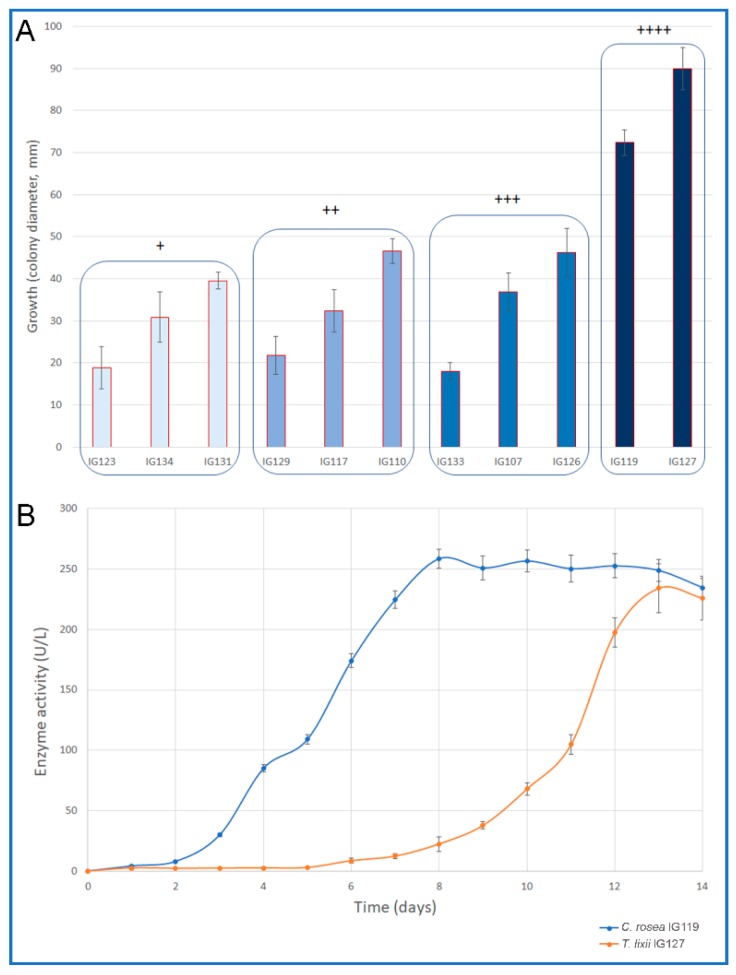
(**A**) growth of marine fungal isolates on the media used for the detection of chitinolytic activity. The isolates have been grouped on the base of their enzyme activity (scores from “+” to “+++”. (**B**) Time course of chitinolytic enzyme production of *Trichoderma lixii* IG127 and *Clonostachys rosea* IG119 on the medium used for the secondary screening.

The results of the preliminary plate screening showed that 11 strains out of 28 (39%) were able to degrade chitin (Table 1). Among them we recorded species of *Chaetomidium, Chaetomium, Clonostachys, Pleospora, Stachybotrys* and *Trichoderma*. Some of them revealed a rather high activity, but the highest activity was recorded for *Trichoderma lixii* IG127 and *Clonostachys rosea* IG119, which showed also best growth (Figure 1A). Various *Trichoderma* species are well known for their high production of hydrolases including high levels of chitinase. Among them it is important to mention *T. resei* for its cellulase production and *T. harzianum* for its very significant chitinolytic activity [28]. *T. lixii* is not known for the production of these enzymes. Nevertheless, this species was recently recognised within the *T. harzianum* complex [29] and its production of chitinolytic enzymes recorded in our study was not unexpected. Various species of *Trichoderma* had been recognised as derived from marine environments also in recent studies [30,31]. 

Additionally, *C. rosea* is known for its production of chitinolytic enzymes [32,33]; as for various *Trichoderma* strains, it is used as a biocontrol agent against phyto-pathogenic fungi due to its mycoparasitic action, which is mainly due to the production of this class of enzymes [33]. The mycoparasitic action of *C. rosea* is so powerful that this species has been defined as the “the best known destructive mycoparasite” [28]. However, only one marine-derived strain of *Clonostachys rosea* has been reported [34], but its chitinolytic or mycoparasitic activities have not been investigated yet. 

### 2.2. Secondary Screening for Chitinolytic Enzymes

The secondary screening in shaken cultures was carried out only for the two selected strains, under non-optimized halophilic condition. Figure 1B shows the time course of the enzyme production for the two strains. The maximum production by *T. lixii* IG127, even if rather good, was strongly delayed (238 U/L after 12 days of incubation) since the fungus showed a long lag phase (5 days). By contrast, *C. rosea* IG119 started its production only after two days of incubation; its maximum activity was recorded at day 8 (258 U/L) to remain quite stable thereafter. The two fungi were selected for further studies. In any case, the maximum activity obtained by the two strains in these non-optimized conditions, even delayed, was at the same level of that recorded for well-known chitinolytic fungi such as *Trichoderma harzianum* P1 and *Lecanicillium muscarium* CFEE 5005 cultivated using similar growth medium, but not in halophilic conditions [20].

### 2.3. Optimal Growth Conditions with Respect to Temperature, pH and Salinity

**Figure 2:**
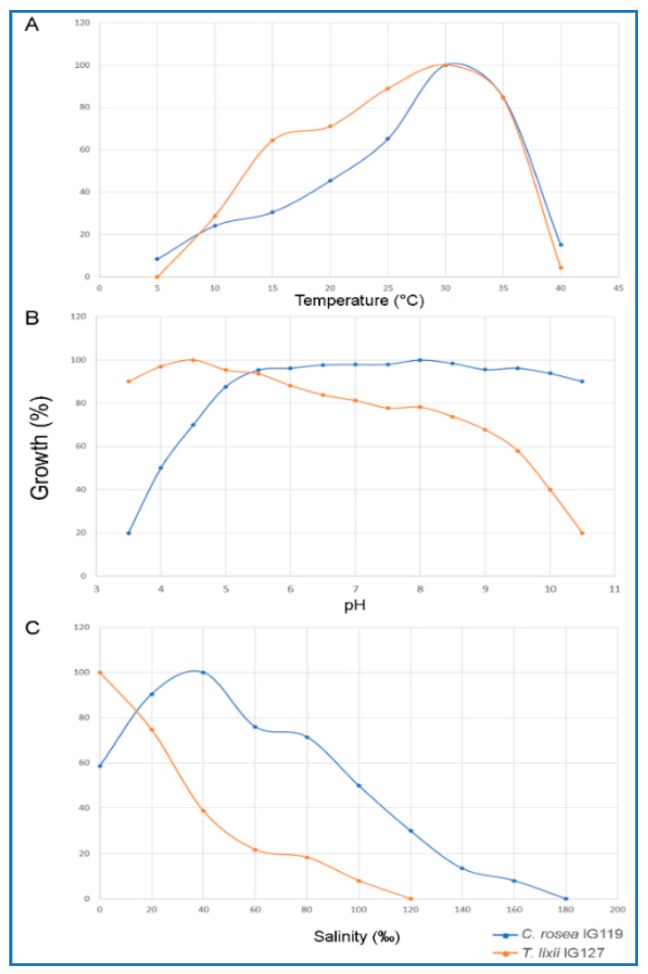
Growth of *Trichoderma lixii* IG127 and *Clonostachys rosea* IG119 in agar media at different values of temperature (**A**), pH (**B**) and salinity (**C**). Data are reported as percentage of maximum growth.

The selected strains were grown on media with different pH (range 3.5–10.5), temperature (range 5–40 °C) and salinity (range 0–180‰) in order to understand their possible adaptation to marine environment (Figure 2).

*T. lixii* IG127 showed to be a mesophilic-psychrotolerant fungus being able to growth in the range 5–40 °C with optimum at 30 °C (Figure 2A). It was also able to grow in a rather wide range of pH (3.5–10.5) with optimum at 4.5 pH (Figure 2B). Its growth with respect to salinity was typical of a slight halotolerant microorganism. Its optimal growth was recorded at about 0‰ of marine salt (growth range 0–100‰ of salinity). However, it is worth noting that its growth rate rapidly dropped with the increase of NaCl concentration; at the sea salinity (38–40‰) growth was already 60% lower than that recorded at 0‰; no growth was recorded at 120‰ and above (Figure 2C). 

For these reasons this strain should not be considered as a marine fungus, but a terrestrial fungus somehow adapted to marine environmental conditions. This would justify the long lag phase recorded in the medium used for the secondary screening, which contained 38‰ of marine salt.

By contrast, *C. rosea* IG119 characteristics suggested that this strain could be considered as a marine fungus. The results obtained evidenced that its optimal conditions for growth appeared to be coherent with the typical environmental parameters of the sea (Figure 2). Best growth was recorded at 8.0 pH (range 3.5–10.5 pH); however, this optimal value must be considered quite virtual since this fungus showed optimal or sub-optimal growth in a very broad range of pH (5.5–9.5) with non-statistically significant variations of its growth rate. Optimal growth in a wide pH range is a known characteristic of this species [28]. In any case, its adaptation to salinity was a decisive point for its classification as a moderate-halophilic marine fungus. Its optimal growth was recorded at 38–40‰ of salinity and a certain euryhalinism was verified since its range of salinity for growth was 0–160‰. The capacity of growth also with minimal concentrations of NaCl was also a worth noting characteristics; in absence of NaCl its growth rate was still 40‰ of that recorded at the optimum. These features were quite unexpected, since it was observed that this species was not able to growth in media containing 50‰ of NaCl [28]. The psychrotolerant behaviour of strain IG119 was less evident since it was not able to grow at 5 °C; however, its eurythermism was somehow very convincing since rather high growth rates were recorded between 15 and 35 °C.

Since this work was aimed to find new high producers of chitinolytic enzymes under halophilic conditions, only *C. rosea* IG119 was selected for the optimization of the growth medium by RSM.

### 2.4. Optimization of the Cultural Medium by Response Surface Methodology 

RSM is one of the most efficient methods to get a performing experimental design. This method is widely used to optimize process parameters in fermentation technology. Additionally, it has also been used to optimize chitinases production by fungi [14,35]. 

In this work RSM was used to optimise the culture medium for best production of chitinolytic activity by *C. rosea* IG119 in view of a possible industrial application where the growth medium composition and its cost have a paramount importance. An industrial growth medium must be as cheap as possible since 20–30% or more of the cost of the final product, obtained by microbial bioprocesses (fermentations), depends on its composition. Moreover, it must permit the maximization of the microbial production, not only in terms of maximum production, but also with respect to yield and/or productivity. In our case the medium used in the preliminary experiments contained, chitin apart, Yeast Nitrogen Base (YNB) that is a very good nitrogen source (essentially a mix of different vitamins, other nitrogen sources and mineral salts) to be used at the laboratory level. However, it is too expensive for industrial applications. By contrast, Corn Steep Liquor (CSL), being a by-product of the corn processing industry, is quite cheap and often used as a nitrogen supplement in industrial fermentations. Due to its very composite formulation rich in carbohydrates, amino acids, polypeptides, fatty acids, other organic compounds and inorganic ions, it is considered a valid bio-stimulant additive for bioprocesses [36,37,38]. Thus, RSM optimization was carried out using an experimental model considering combinations of different amounts of these two substrates according to a D-optimal design, as suggested by the software.

Model performance was definitely good. Data were best fitted by a polynomial quadratic equation, as it can be inferred by the good agreement of experimental data with those estimated by the model. Table 2 shows the regression results of the experimental data of the D-optimal model, which revealed that the effect of CSL was more significant than that of YNB on both production and productivity of the chitinolytic enzymes by *C. rosea*.

In addition, the model revealed high reliability and good statistical performance. For both the analysed responses (production and productivity), the probability for the regression was significant at ≥95% and there was no lack of fit. 

As for the chitinolytic enzyme production and productivity, the correlation coefficients (R^2^), indicating the fraction of response variation explained by the model, were very high (0.959 and 0.95, respectively). This means that the statistical model can explain 95.9 and 95.0% of the response variability. Moreover, Q^2^ values, indicating the fraction of response variation that can be predicted by the model, were rather good (0.92 and 0.895, respectively). Finally, the results of the ANOVA analysis run by the Modde 5 software showed significant F-values (60.74 and 49.66, respectively, P = 0.000), indicating that the model terms were quite significant (Table 2). 

**Figure 3:**
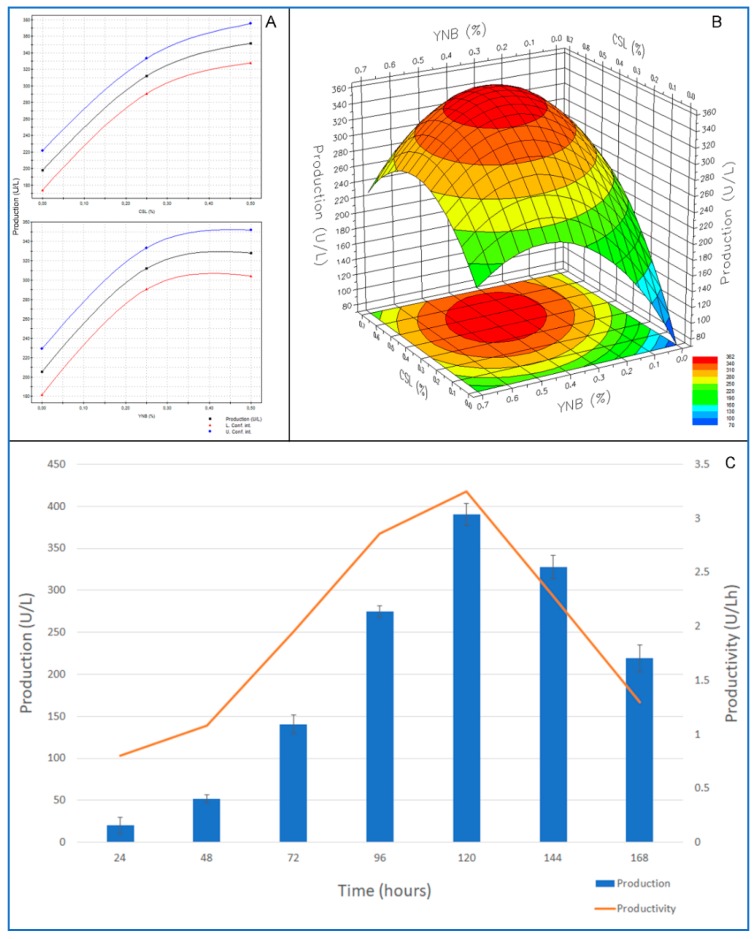
Single (**A**) and combined (**B**) effects of different Corn Steep Liquor (CSL) and Yeast Nitrogen Base (YNB) concentrations on the chitinolytic enzyme production by *Clonostachys rosea* IG119 as reported by the model. Time course of enzyme production and productivity by *Clonostachys rosea* IG119 cultivated on the optimized medium suggested by the Response Surface Methodology (RSM) model (**C**).

The RSM permitted taking into account the effects of the two nitrogen sources as used alone (Figure 3A). YNB appeared to be somehow inhibiting when its concentration exceeded 0.4%, while the enzyme activity seemed to be directly proportional to CSL concentration, even if the trend showed that a plateau could be reached rapidly over 0.5%. However, best enzyme production was obtained by a synergistic combination of the two substances. Model indicates that highest production (circa 360 U/L) could be obtained at about 0.35% of YNB and 0.45% CSL (Figure 3B). As for productivity, a maximum of circa 3 U/Lh was predicted at the same concentrations of the two nitrogen sources (figure not shown). However, the best result obtained experimentally was 363.8 U/L using 0.5% of YNB and 0.5% CSL (Experiment N° 4) after 120 h of incubation. By running the software “optimizer” function, the model suggested that optimal conditions for the enzyme production were CSL 0.47%, YNB 0.37% in order to expect a maximum activity of 365.3 U/L. The experimental validation of these expected optimal conditions, showed that actual maximum production and productivity were definitely higher than those predicted (394 U/L and 3.3 U/Lh, respectively, at 120 h): Activity under optimized conditions was increased circa 1.53-fold.

It is worth noting that, the maximum activity obtained with the RSM optimization was much higher (circa 53%) than that recorded during the secondary screening. In addition, maximum production was obtained after only 120 h (3 days before!).

While strain IG119 chitinolytic activity seems to be quite high, the comparison with other strains of *C. rosea* is not possible since, to the best of our knowledge, there is no available study investigating the ability of the species to produce this class of enzymes in shaken cultures. While the species is known for the production of this class of enzymes, only experiments carried out in plate cultures are reported in literature, supplying just qualitative or semi-quantitative indication of the enzyme activity. By contrast, comparison with the production of other fungi, known for their high chitinolytic activity (i.e., *Trichoderma* spp., *Penicillium* spp. and *Lecanicillium* spp.) is possible even if not easy due to the huge amount of publications available and, in particular, for the different approaches related both to the detection and to the expression of the enzyme activity; actually, many authors use a variegated pattern of arbitrary units instead of the conventional International Unit definition.

In a previous early study, we investigated two selected strains of *T. harzianum* (P 1 and T 22), known for their high production of chitinolytic enzymes, in comparison with a new promising Antarctic strain of *Lecanicillium muscarium* (ex. *Verticillium lecanii*) [9,20]. The activity of the three strains, tested using culture conditions very similar to those reported in the present study, was comparable to that of *C. rosea* IG119 prior to perform RSM optimization (circa 250 U/L after 72 h of growth). Subsequent optimization of *L. muscarium* chitinolytic enzymes production in bioreactor by RSM lead to increase it activity up to circa 380 U/L [9,35]. This value is similar to that obtained here in shaken flasks after RSM. Moreover, strain IG199 activity appeared much higher than that (circa 200 U/L) of another well-known strain (CECT 2413) of *T. harzianum,* studied in early work by De la Cruz et al. [39]. 

Another very promising fungus formerly investigated in our laboratory, *Penicillium janthinellum* P9, showed a better performance than *C. rosea* IG119; it enzyme activity in shaken cultures was circa 400 U/L prior to optimization (both in shaken cultures and bioreactor by RSM) that brought it up to circa 650 U/L [40,41].

Recently, a strain (ITCC-10,364.16) of *Humicola grisea* was investigated, during a screening of soil microorganisms, for its “notable” chitinolytic activity. However, after RSM optimization of its culture media, its activity was barely higher than 180 U/L [42]. 

One of the most interesting organisms investigated in recent years was the fungus *Duddingtonia flagrans*, which was able to produce circa 1030 U/L, after RSM optimization of its cultural medium. The results of the mentioned paper indicated this species as one of the most potent chitin-degrader organism among prokaryotes [43].

On the whole, even if much extensive comparison could be performed, it would be possible to affirm that the production of chitinolytic enzymes by *C. rosea* IG119 is high enough to consider the fungus as a promising high producer for possible applications, particularly when halophilic conditions are required. 

## 3. Materials and Methods

### 3.1. Collection of Samples and Isolation of Pure Cultures of Fungi 

Fungi were isolated from the marine phanerogam *Posidonia oceanica* and its epiphytic algae *Dictyota dichotoma* (Pheophyta) and *Sphaerococcus coronopifolius* (Rhodophyta) collected by scuba divers using sterile containers. Isolation of microorganisms was carried within two hours from sample retrieval as reported by [44]. Pure cultures of the fungal isolates were cryogenically maintained at −40 °C in the culture collection of microorganisms of the “Laboratorio di Ecologia dei Funghi Marini”, DEB, University of Tuscia. Strains had been revitalized and sub-cultured on Malt Extract Agar (MEA, Difco, Detroit, MI, USA) added with 38‰ of marine salt.

### 3.2. Strain Identification

All isolates were preliminary classified on the basis of specific taxonomic keys, according to macroscopic, microscopic and physiological features [28,45,46,47,48,49,50]. The two possible high producers, selected for further investigations, were classified also by molecular methods using the following targets: ITS region (ITS1-5.8S-ITS2) of rDNA for both strains; the actin gene for IG 127 only. Genomic DNA was extracted using ZR Fungal/Bacterial DNA MiniPrep Kit (Zymo Research, Irvine, CA, USA) according to the manufacturer’s directions. The ITS region was amplified using the universal primers ITS5 and ITS4 [51] and the actin gene with ACT512F and ACT783R [52]. Taxonomic assignments were inferred by querying with the BLASTn algorithm hosted at NCBI, similarity values higher than 98% (e-value > e-100) were considered reliable and the results were confirmed morphologically. The taxonomic position was inferred through phylogenetic analysis if low sequence similarity (<98%) was obtained.

### 3.3. Screening Procedure

Semi-quantitative test for chitinolytic enzyme production (plate screening) was carried out as reported previously with slight modifications [1]. Briefly: Pre-poured MEA plates, containing 3% of colloidal chitin and 38‰ NaCl, were inoculated with punctiform inocula, picking mycelia with sterile needles from 5–7 days cultures grown on MEA slants (at 25 °C). To avoid any interference from nearby colonies, only one isolate was inoculated onto each plate (90 mm diameter). Activity was arbitrarily scored, based on the diameter of clarification halos on the plates, as follows: “-”, no halos; “+”, halos between 0.1 and 10.0 mm; “++”, halos between 10.1 and 30.0 mm; “+++”, halos between 30.1 and 60.0 mm and “++++”, halos between 60.1 and 90.0 mm.

Halos of chitin degradation were recorded (daily for 7 days) and measured by an image analysis software (I.A.S. ver. 008 000, Delta Sistemi, I). Secondary screening in liquid cultures was carried out for the selected strains (*Trichoderma lixii* IG127 and *Clonostachys rosea* IG119) in shaken cultures on a medium [35] containing 1% of colloidal chitin and Yeast Nitrogen Base (YNB, Difco, Detroit, MI, USA) added with Marine salt to a final concentration of 38‰. Erlenmeyer flask (500 mL) containing 100 mL of medium were inoculated with circa 2.5 mg/mL dry weight of mycelium grown for 5 days on Malt Extract Broth (MEB, Difco, Detroit, MI, USA) and incubated at 25 °C and 250 rpm for 336 h. Samples for chitinolytic activities were withdrawn daily. All media were autoclaved at 121 °C.

### 3.4. Optimization of Growth Conditions of pH, Temperature and Salinity 

The selected strains were tested in for their optimal growth at different pH (range 3.5–10.5, steps of 0.5 pH), temperatures (range 5–40 °C, steps of 0.5 °C) and salinity (range 0–180‰, steps of 20‰; tests were also carried out at 38‰, the sea salinity recorded at the sampling site) on MEA plates modified to adjust pH and salinity. For each strain pH optimization was carried at 25 °C and 38‰ marine salt; temperature optimized at optimal pH and 3.8‰ marine salt, while salinity was optimized at optimal pH and temperature.

### 3.5. Optimization of Culture Medium by RSM Factorial Design

Effect of the combined action of Corn Step Liquor (CSL, Sigma-Aldrich, St. Louis, MO USA) and YNB on the production and productivity of chitinolytic activity was optimised by a D-optimal design, with the following independent variables (factors):

X_1_ = CSL (g/L)

X_2_ = YNB (g/L)

The above dimensional independent variables were coded as dimensionless terms by the following equation:

X*_i_* = (A*_i_* − A*_0_*)/ΔA      *i* = 1, 2

where X*_i_* is a coded value and A*_i_* is the actual value of the variable, A*_0_* is the actual value of the same variable at the centre point and ΔA is the variable step change.

The range of the variables is given in Table 3. 

Data were subjected to analysis of variance (ANOVA) and fitted according to a second-order polynomial model shown by:

*Y* = βo + ∑β_i_*X*_i_ + ∑β_ii_*X*_i_^2^ + ∑β_ij_*X*_i_*X*_j_

where *Y* is the predicted response variable, βo is the intercept, βi and βii are the linear coefficient and quadratic coefficient, respectively, βij is the interaction coefficient and *X*i and *X*j are the coded forms of the input variables. To estimate the impact of single independent variables on the response, regardless of the presence of the other factors, main effects were calculated using:

*Y* = βo + β_i_*X*_i_ + β_ii_*X*_i_^2^

Statistical examination of results and response surface study were carried out by the MODDE 5.0 software (Umetrics AB, Sweden). 

Fermentations under optimal conditions for best enzyme production, as suggested by the model, were carried out in triplicate in subsequent experiments. All media for the RSM optimization were inoculated as mentioned above.

### 3.6. Analytical Methods

For the secondary screening and RSM optimization the overall chitinolytic activity was determined by the method of dinitrosalicylic acid (DNSA), using N-Acetyl-D-glucosamine for standard curve, as previously reported [40,41]. 

Under the assay conditions, one unit (U) of enzyme activity was defined as the amount of enzyme which released 1 µmol per mL per min.

## 4. Conclusions

*Clonostachys rosea* IG119 was able to release high levels of chitinolytic enzymes under halophilic conditions. The results of this study indicate that this marine fungus could be considered as a new promising organism for possible applications in biotechnology. The RSM optimization allowed the increase of *C. rosea* enzyme activity (circa 1.53-fold enhancement) to levels suitable for possible industrial production. This work supplied the first demonstration that this fungal species also contains strains specifically adapted to the marine environment. In addition, it was the first study reporting high chitinolytic enzymes production by a marine strain of *C. rosea*. 

## Figures and Tables

**Table 1:** Taxonomical affiliation and chitinolytic enzyme activity of the various fungal strains isolated from different marine substrates.

Strain	Taxa	Substrate	Activity
IG132	*Alternaria chlamydospora*	DD	−
IG135	*Arthrinium* sp.	SC	−
IG133	*Aspergillus flavus*	SC	+++
IG105	*Aspergillus insuetus*	PO	ng
IG136	*Aspergillus spelaeus*	SC	ng
IG125	*Aspergillus versicolor*	DD	−
IG118	*Cephalotrichum gorgonifer*	PO	−
IG129	*Chaetomidium fimeti*	DD	++
IG134	*Chaetomidium fimeti*	SC	+
IG110	*Chaetomium* sp. 1	PO	++
IG107	*Chaetomium* sp. 2	PO	+++
IG124	*Cladosporium* sp. 1	DD	−
IG101	*Cladosporium* sp. 2	PO	−
IG123	*Clonostachys rosea*	PO	+
IG117	*Clonostachys rosea*	PO	++
IG119	*Clonostachys rosea*	PO	++++
IG120	*Fusarium* sp.	PO	ng
IG100	*Mariannaea* sp.	PO	ng
IG121	*Microascus brevicaulis*	PO	ng
IG103	*Penicillium* sp.	PO	−
IG113	*Pleospora* sp.	PO	ng
IG122	*Scopulariopsis* sp.	PO	−
IG126	*Stachybotrys chlorohalonatus*	DD	+++
IG127	*Trichoderma lixii*	DD	++++
IG131	*Micelia sterilia* 1	DD	+
IG114	*Micelia sterilia* 2	PO	ng
IG108	*Micelia sterilia* 3	PO	ng
IG115	*Micelia sterilia* 4	PO	ng

Legend: DD = *Dictyota dichotoma*, PO = *Posidonia oceanica*, SC = *Sphaerococcus coronopifolius*. ng = no growth. Activity was arbitrary scored from “−“ no activity to “++++” highest activity.

**Table 2:** Model coefficients estimated by multiple linear regression (significance of regression coefficients).

	**Production (U/L)**			**Productivity (U/Lh)**		
**Coefficient**	**RC**	**SE**	**P**	**RC**	**SE**	**P**
Constant	3.5158	0.1112	1.10 × 10^−^^13^	3.4515	0.1224	4.7937 × 10^−13^
CSL	0.8666	0.0688	1.17 × 10^−8^	0.9656	0.0758	1.0124 × 10^−8^
YNB	0.6908	0.0688	1.72 × 10^−7^	0.5917	0.0758	2.9116 × 10^−6^
CSL*CSL	−0.4204	0.1139	2.71 × 10^−3^	−0.1911	0.1254	1.5150 × 10^−1^
YNB*YNB	−0.5110	0.1139	6.13 × 10^−4^	−0.5601	0.1254	6.3504 × 10^−4^
CSL*YNB	−0.2251	0.0843	1.92 × 10^−2^	−0.0978	0.0928	3.1117 × 10^−1^
**ANOVA table (Modde 5)**						
F value	60.7442	P = 0.000		49.656	P = 0.000	
Q^2^	0.92			0.895		
R^2^	0.959			0.95		
R^2^ adjusted	0.943			0.931		

**Legend.** RC = regression coefficient, SE = standard error, P = *p* value.

**Table 3:** Experimental setup combining different concentration of CSL and YNB as suggested by the model.

Experiment	CSL (%)	YNB (%)
N1, N12	0	0
N2, N13	0.5	0
N3, N14	0	0.5
N4, N15	0.5	0.5
N5, N16	0	0.25
N6, N17	0.5	0.25
N7, N18	0.25	0
N8, N19	0.25	0.5
N9, N10, N11	0.25	0.25
